# Supplementary material for: Trends in Net Survival from Vulvar Squamous Cell Carcinoma in Italy (1990–2015)
Source: J Clin Med. 2023 Mar 10;12(6):2172. doi: 10.3390/jcm12062172 (PMC10054662; doi:10.3390/jcm12062172)
Supplement: Supplementary file 1 [file jcm-12-02172-s001.zip › jcm-2197395-supplementary.pdf]

Trends in net survival from vulvar squamous cell carcinoma in Italy (1990-2015)

Silvia Mancini [1] et al.

[1] Romagna Cancer Registry, Romagna Cancer Institute (IRCCS Istituto Romagnolo per lo Studio dei Tumori (IRST) Dino Amadori), 47014 Meldola, Forlì, Italy; silvia.mancini@irst.emr.it

Supplementary Figure S1.

Cancer Registry	1990	1991	1992	1993	1994	1995	1996	1997	1998	1999	2000	2001	2002	2003	2004	2005	2006	2007	2008	2009	2010	2011	2012	2013	2014	2015
**North**																										
Bergamo																		2007	2008	2009	2010	2011	2012			
Biella						1995	1996	1997	1998	1999	2000	2001	2002	2003	2004	2005	2006	2007	2008	2009	2010					
Brescia										1999	2000	2001	2002	2003	2004	2005	2006	2007	2008	2009	2010	2011	2012	2013		
Cremona																2005	2006	2007	2008	2009	2010	2011	2012			
Ferrara		1991	1992	1993	1994	1995	1996	1997	1998	1999	2000	2001	2002	2003	2004	2005	2006	2007	2008	2009	2010	2011				
Friuli Venezia Giulia						1995	1996	1997	1998	1999	2000	2001	2002	2003	2004	2005	2006	2007	2008	2009	2010					
Genova	1990	1991	1992	1993	1994	1995	1996	1997	1998	1999	2000	2001	2002	2003	2004	2005	2006	2007	2008	2009	2010	2011				
Mantova										1999	2000	2001	2002	2003	2004	2005	2006	2007	2008	2009	2010	2011	2012	2013	2014	
Modena	1990	1991	1992	1993	1994	1995	1996	1997	1998	1999	2000	2001	2002	2003	2004	2005	2006	2007	2008	2009	2010	2011	2012	2013		
Monza-Brianza																		2007	2008	2009	2010	2011	2012			
Parma	1990	1991	1992	1993	1994	1995	1996	1997	1998	1999	2000	2001	2002	2003	2004	2005	2006	2007	2008	2009	2010	2011	2012	2013	2014	2015
Pavia														2003	2004	2005	2006	2007	2008	2009	2010	2011	2012			
Reggio Emilia							1996	1997	1998	1999	2000	2001	2002	2003	2004	2005	2006	2007	2008	2009	2010	2011	2012	2013	2014	2015
Romagna	1990	1991	1992	1993	1994	1995	1996	1997	1998	1999	2000	2001	2002	2003	2004	2005	2006	2007	2008	2009	2010	2011	2012	2013	2014	2015
South Tyrol						1995	1996	1997	1998	1999	2000	2001	2002	2003	2004	2005	2006	2007	2008	2009	2010	2011	2012			
Trento						1995	1996	1997	1998	1999	2000	2001	2002	2003	2004	2005	2006	2007	2008	2009	2010	2011	2012			
Torino	1990	1991	1992	1993	1994	1995	1996	1997	1998	1999	2000	2001	2002	2003	2004	2005	2006	2007	2008	2009	2010	2011	2012			
Varese	1990	1991	1992	1993	1994	1995	1996	1997	1998	1999	2000	2001	2002	2003	2004	2005	2006	2007	2008	2009	2010	2011	2012			
Veneto	1990	1991	1992	1993	1994	1995	1996	1997	1998	1999	2000	2001	2002	2003	2004	2005	2006	2007	2008	2009	2010			2013		
**Centre**																										
Firenze Prato	1990	1991	1992	1993	1994	1995	1996	1997	1998	1999	2000	2001	2002	2003	2004	2005	2006	2007	2008	2009	2010					
Macerata		1991	1992	1993	1994	1995	1996	1997	1998	1999	2000	2001														
Umbria					1994	1995	1996	1997	1998	1999	2000	2001	2002	2003	2004	2005	2006	2007	2008	2009	2010	2011	2012	2013	2014	2015
Viterbo																	2006	2007	2008	2009	2010	2011	2012			
**South**																										
Agrigento																						2011	2012	2013		
Barletta Andria Trani																	2006	2007	2008	2009	2010	2011	2012	2013		
Basilicata																	2006	2007	2008	2009	2010					
Benevento																					2010	2011	2012	2013		
Brindisi																	2006	2007	2008	2009	2010					
Catania-Messina-Enna														2003	2004	2005	2006	2007	2008	2009	2010	2011	2012	2013		
Catanzaro														2003	2004	2005	2006	2007	2008	2009	2010					
Lecce														2003	2004	2005	2006	2007	2008	2009						
Napoli							1996	1997	1998	1999	2000	2001	2002	2003	2004	2005	2006	2007	2008	2009	2010	2011	2012	2013	2014	2015
Palermo														2003	2004	2005	2006	2007	2008	2009	2010	2011	2012	2013	2014	2015
Ragusa-Caltanissetta	1990	1991	1992	1993	1994	1995	1996	1997	1998	1999	2000	2001	2002	2003	2004	2005	2006	2007	2008	2009	2010	2011	2012	2013		
Salerno							1996	1997	1998	1999	2000	2001	2002	2003	2004	2005	2006	2007	2008	2009	2010	2011	2012			
Sassari			1992	1993	1994	1995	1996	1997	1998	1999	2000	2001	2002	2003	2004	2005	2006	2007	2008	2009	2010	2011				
Siracusa										1999	2000	2001	2002	2003	2004	2005	2006	2007	2008	2009	2010	2011	2012	2013		
Trapani													2002	2003	2004	2005	2006	2007	2008	2009	2010	2011				

Figure S1. Years of data contribution by the 38 cancer registries participating in the study on time trends in net survival from vulvar squamous cell carcinoma in Italy between 1990 and 2015.
